# Hp-Positive Chinese Patients Should Undergo Colonoscopy Earlier and More Frequently: The Result of a Cross-Sectional Study Based on 13,037 Cases of Gastrointestinal Endoscopy

**DOI:** 10.3389/fonc.2021.698898

**Published:** 2021-08-26

**Authors:** Cheng Wang, Junbin Yan, Beihui He, Shuo Zhang, Sumei Xu

**Affiliations:** ^1^Applied Math Department, China Jiliang University, Hangzhou, China; ^2^The First Affiliated Hospital of Zhejiang Chinese Medical University, Hangzhou, China; ^3^Department of Gastroenterology, The First Affiliated Hospital of Zhejiang Chinese Medical University, Hangzhou, China; ^4^Department of General Practice, The First Affiliated Hospital of Zhejiang Chinese Medical University, Hangzhou, China

**Keywords:** colorectal polyps, colorectal cancer, adenoma, cross-sectional study, age, Chinese

## Abstract

**Background:**

In China, the prevalence and mortality of colorectal cancer (CRC) have always been high, and more than 95% of CRC cases have evolved from colorectal polyps (CPs), especially adenoma. Early detection and treatment of CPs through colonoscopy is essential to reduce the incidence of CRC. Helicobacter pylori (Hp) is regarded as a risk factor for gastritis and gastric cancer and may also be a risk factor for CPs and CRC. However, few studies based on vast clinical cases exist in China to clarify whether Hp is a risk factor for CPs and CRC, and whether Hp-positive patients need to undergo colonoscopy checks earlier. This article attempts to make up for that deficiency.

**Method:**

This cross-sectional study was conducted based on 13,037 patients without a treatment history of Hp who underwent their first gastroscopy and colonoscopy simultaneously at The First Affiliated Hospital of Zhejiang Chinese Medical University from January 2018 to December 2019. Pearson χ^2^ test and logistic regression were used to determine whether Hp is a risk factor for CPs and CRC. Multifactor analysis of variance was used to define the impact of Hp on CPs prevalence with different ages, sexes.

**Results:**

For Chinese individuals, Hp is a risk factor for CPs and CRC. The odds ratio (OR) value are 1.228 (95% CI, 1.130 to 1.336) and 1.862 (95% CI 1.240-2.796), respectively. Hp-positive patients have a higher probability of multiple or large intestinal polyps. However, Hp infection does not increase the incidence of adenomas, nor does it affect the pathological type of adenomas. The OR of Hp on the risk of CPs was 1.432 (95%CI 1.275-1.608) for males but increased to 1.937 (95%CI 1.334-2.815) for those aged 35 to 40. For females, the results were similar.

**Conclusions:**

For the Chinese, Hp is a risk factor for CPs and CRC (OR>1); the infection of Hp increased CPs risk in Chinese of all ages, especially aged 35-40, suggesting that Hp-positive patients should undergo colonoscopy frequently.

## Introduction

The incidence of colorectal cancer (CRC) ranks second worldwide among malignant tumors and increases annually. Each year, nearly 900,000 people die from CRC (1). From 2006 to 2016, CRC incidence in China increased by 34% (2), ranking 5th in malignant tumors. Colorectal polyps (CPs) are precancerous lesions related to the occurrence of CRC (3).

CPs are conventionally divided into nonneoplastic hyperplastic polyps and adenomas; the latter is the culprit in CRC development. Compared with larger polyps (≥ 10mm), small polyps (6-9mm) and micropolyps (1-5mm) generally do not have the characteristics of advanced adenomas and are less likely to be CRC. In addition, patients with multiple polyps also have a higher risk of CRC. In most cases, CPs have no apparent symptoms. Therefore, screening risk factors associated with CPs and CRC, which are applicable for Chinese patients, will help early diagnosis and prevention of the diseases.

Helicobacter pylori (Hp) is recognized as the main pathogenic factor of peptic ulcer, atrophic gastritis, and gastric cancer (4). In recent years, with the deepening of Hp research, it was found that Hp infection may be related to the occurrence and progression of CPs and CRC (5–7). However, most related studies are meta-analyses and lack direct verification through clinical patients. In addition, large-scale sample research on Chinese cases is still scarce. It is crucial to make up for this deficiency. This study conducted a cross-sectional analysis of 13,037 Chinese patients without a history of Hp treatment who underwent gastrointestinal endoscopy at The First Affiliated Hospital of Zhejiang Chinese Medical University from 2018.1-2019.12 to clarify whether Hp is a risk factor for CPs and CRC, and whether Hp-positive Chinese patients need to undergo colonoscopy screening earlier.

## Methods

### Objects

This study was approved by the Medical Ethics Committee of The First Affiliated Hospital of Zhejiang Chinese Medical University and waived patient informed consent (2021-K-339-01). Because this study was retrospective, we followed the Observational Studies in Epidemiology (STROBE) reporting guidelines (8).

**Figure 1:**
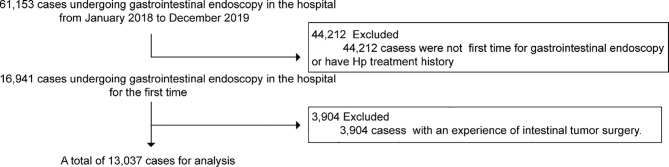
Flowchart of cases selection.

Gastrointestinal endoscopy, pathology, biopsy, and immunohistochemical analysis data of 61,153 inpatients, outpatients, and physical examinees in Hubin and Xiasha districts of The First Affiliated Hospital of Zhejiang Chinese Medical University from January 2018 to December 2019 were collected. Patients who were not undergoing gastrointestinal endoscopy simultaneously in the hospital for the first time or with the experience of intestinal tumor surgery or Hp treatment history were excluded. A total of 13,037 cases were selected for analysis (**Figure 1**). HE staining of the gastric antrum and gastric mucosa specimens was used to detect Hp infection.

### Analyzing the Relationship Between Hp and CPs, CRC

① Among the 13,037 cases, 3,291 cases were Hp-positive, and 9,746 cases were Hp-negative. According to disease classification, the above 13,037 cases were divided into three groups, the noncolorectal polyps group (NCPs) (7,968 cases), CPs group (4,969 cases), and CRC group (100 cases) to analyze the correlation between Hp, CPs, and CRC. ② A total of 4,435 cases that had undergone biopsy and histopathological analysis, were selected from 4,969 CPs cases to investigate the correlation between Hp and the size, morphological and pathological characteristics of polyps. ③ A total of 2,508 cases diagnosed as adenoma, a type of intestinal polyp that is highly prone to CRC, were chosen to examine the correlation between Hp and the type and grade of adenoma. ④ A total of 375 cases, which underwent immunohistochemical analysis, were selected to investigate the correlation between Hp and P53, Ki-67, MLH1, PMS2, MSH2, and MSH6. The immunohistochemical EnVision method was used to detect the P53, Ki-67, MLH1, PMS2, MSH2, and MSH6 expressions of CPs and then group them. According to the proportion of positive cells (9), P53 was divided into 5 grades: (1) ≤5%; (2) 6% to 25%; (3) 26% to 50%; (4) 51-75%; and (5) greater than 76%. Ki-67 was recorded as positive when it was equal to or greater than 30%. The criteria for the positive results of mismatch repair genes MLH1, PMS2, MSH2, and MSH6 were if the expression of the mismatch repair gene protein in the nucleus was determined to be positive (independent of the strength of nuclear staining) or the diffuse or focal distribution of positive cells (10).

### Analyzing the Relationship Between Different Hp Infection Levels and CPs, CRC

Cases in the Hp-positive group will be further divided into three different degrees of Hp (+), (++), and (+++), according to the results of HE staining. The classification of Hp infection is based on the number of Hp bacteria in a field of view under the microscope. The specific category refers to the criterion of Consensus Opinions on Chronic Gastritis in China (2012, Shanghai) (11). +: A few Hp bacteria; Hp distribution is less than 1/3 of the total length of the specimen. ++: Continuous and sparsely existing on the surface of the specimen; Hp distribution reaches or exceeds 1/3 of the entire length of the specimen, less than 2/3. +++: Hp exists in piles, and is basically distributed over the entire length of the specimen. The prevalence of CPs and CRC at different Hp infection levels was calculated to judge whether different Hp infection levels affect the incidence of CPs and CRC.

### Statistical Analysis

SPSS 22.0 statistical software was used for statistical analysis. The measurement data are shown as the mean ± standard deviation; The counting data are represented by the number of cases (percentage). The comparison between groups adopted the Pearson χ^2^ test and Wilcoxon rank sum test. Logistic regression was used to determine the OR value of Hp, age, and gender for CPs, adenoma, CRCs, and corresponding pathological results. Multivariate analysis of variance was used to study the interaction of Hp infection and age on CPs. *P <*0.05 indicates that the result is statistically significant.

## Results

### Analysis of Influencing Factors of Hp

Among the total of 13,037 cases, 9,746 cases were Hp-negative, and 3,291 cases were positive *via* gastroscopy biopsy and HE staining. The rate of Hp infection is 25.2%. This rate is far below the 55.8% Hp infection rate reported in literature in China (12). The infection rate of Hp is closely related to the environment and diet (13). Studies have found that HP can be detected in unclean water (14). The vast majority of subjects in the study came from Zhejiang. Zhejiang is a province in China with good economic conditions. Compared with economically underdeveloped areas, Zhejiang has better clean food and public health. Therefore, it is understandable that the Hp infection rate in the study is far lower than the Hp infection rate reported in the Chinese literature. The age of 9,746 Hp-negative cases was 47.0 ± 12.8, including 4,773 males and 4,973 females. A total of 3,291 Hp-positive cases included 1,737 males and 1,554 females, aged 50.7 ± 12.0 years old. Male incidence was 26.7%, and female incidence was 23.6%. The χ^2^ test showed that the Hp infection rate in men was higher than that in women (*P* < 0.01) (**Table 1**), indicating that compared with females, males may be more susceptible to being infected by Hp.

**Table 1:** Analysis of Hp infection.

Group		Numbers (male/female)		Age Mean ± SD	
Hp-negative		9746 (4773/4973)		47.0 ± 12.8	
Hp-positive		3291 (1737/1554)		50.7 ± 12.0	
Total		13037 (6510/6527)		51.8 ± 12.6	
**The χ^2^ test for the effect of HP and Gender**					
**Factor**		**Hp (negative) n=9746**	**Hp (positive) n=3291**	**χ^2^**	***P* value**
Gender	Male	4773 (49.0%)	1737 (52.8%)	14.258	0.000**
	Female	4973 (51%)	1554 (47.2%)		

**P < 0.01.

### Analysis of Influencing Factors of CPs and CRC

In the 13,037 cases, there were 7,968 cases in the NCPs group, 4,969 cases with CPs, and 100 cases with CRC. General information was as follows: In the CPs group, there were 2,897 males and 2,072 females (1.40:1), and the age ranged from 17 to 90 years old (50.6 ± 11.11). There were 61 males and 39 females in the CRC group with a male-to-female ratio of 1.56:1, and age from 28 to 79 years old (61.3 ± 10.8). The 7,968 cases in the NCPs group included 4,416 males and 3,552 females. The ratio was 1.24:1 (male- to-female); the mean age was 49.3 ± 12.8. The total prevalence of CPs and CRC was 38.11% and 7.67‰, respectively. According to the results of χ^2^ test, sex, and age both had statistically significant impacts on the incidence of CPs and CRC (*P* < 0.01) (**Table 2**).

**Table 2:** Analysis of CPs and CRC.

Group		Numbers (male/female)			Age mean ± SD	
CPs		4969 (2897/2072)			50.6 ± 11.11	
CRC		100 (61/39)			61.3 ± 10.8	
**The χ^2^ test for the effect of Gender, Age on CPs and CRC**						
**Factor**		**NCP n=7968**	**CPs n=4969**	**CRC n=100**	**χ^2^**	***P* value**
Gender	Male	3552 (44.6%)	2897 (58.3%)	61 (61.0%)	235.50	0.000**
	Female	4416 (55.4%)	2072 (41.7%)	39 (39.0%)		
Age (year)	<45	2824 (35.4%)	760 (15.3%)	6 (6.0%)	645.97	0.000**
	≥45	5144 (64.6%)	4209 (84.7%)	94 (94.0%)		

**P < 0.01.

### The Relationship Between Hp, CPs, and CRC

After statistical analysis, it was found that, among the 9,746 Hp-negative cases, there were 3,609 cases with CPs. The prevalence of CPs was 37.0%. Among 3,291 Hp-positive cases, there were 1,360 cases with CPs. The prevalence rate was 41.3%. The results of χ^2^ test indicated a significant correlation between Hp infection and CPs (*P* < 0.01). Of the 13037 total cases, 9,746 were Hp-negative, including 62 cases of CRC accounting for 6.36‰. There was a total of 3,291 Hp-positive cases, among which 38 had CRC, accounting for 11.54‰. The χ2 test showed a significant correlation between Hp and CRC incidence (*P* < 0.01). The results indicated that Hp-positive cases had a higher prevalence of CPs and CRC (**Table 3**).

**Table 3:** Analysis of CPs and CRC.

Group		CPs (%)			CRC (%)	
Hp-negative		3609 (37.0%)			62 (0.64%)	
Hp-positive		1360 (41.3%)			38 (1.15%)	
Total		4969 (38.1%)			100 (0.77%)	
**The χ^2^ test for the effect of Hp, Gender and Age on CPs and CRC**						
**Factor**		**NCP n=7968**	**CPs n=4969**	**CRC n=100**	**χ^2^**	***P* value**
Hp	negative positive	6075	3609	62	29.850	0.000**
		1893	1360	38		

**P < 0.01.

**Figure 2:**
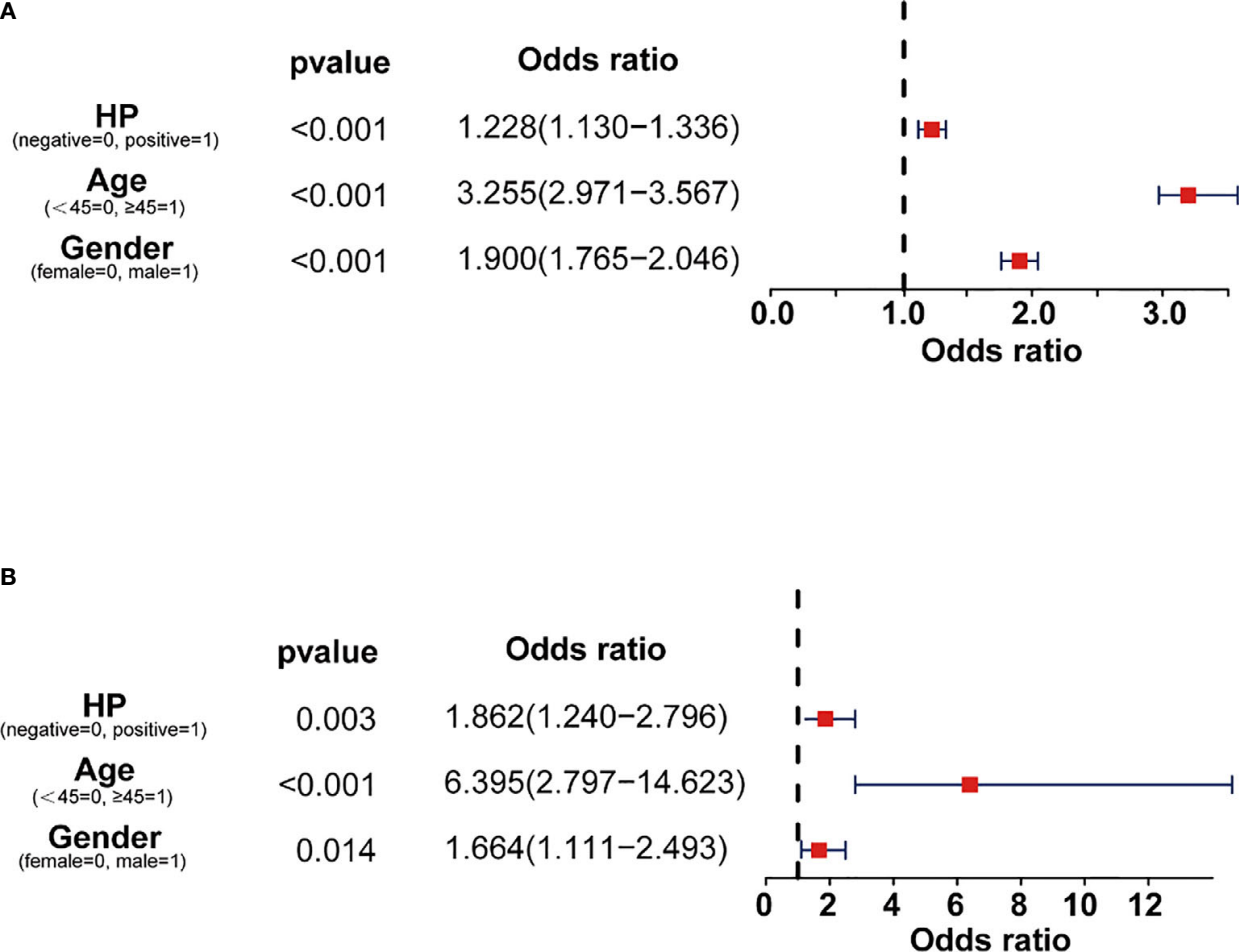
OR value of Hp infection from logistic regression. **(A)** OR value of gender, age, and Hp for CPs. **(B)** OR value of gender, age, and Hp for CRC.

Multivariate logistic regression was used to analyze whether Hp infection is a risk factor for CPs and CRC. The results showed that the OR value of Hp with CPs was 1.228 (95%CI 1.130-1.336), indicating that Hp infection could promote the occurrence of CPs (**Figure 2A**). The OR value for Hp with CRC was 1.862 (95%CI 1.240-2.796), suggesting that Hp infection will increase CRC incidence (**Figure 2B**).

### The Relationship Between Hp and the Size, and Number of CPs

Among the 4,969 cases with CPs, 3,609 cases were Hp negative, of which 503 cases were CPs with a diameter ≥ 10 mm (13.9%), and 3106 cases were CPs with a diameter of < 10 mm (86.1%). Multiple polyps (n ≥ 2) were found in 1,480 cases (41.0%), and the single polyp (n < 2) was found in 2,129 cases (59.0%). A total of 1,360 Hp positive cases included 224 cases with CPs diameter ≥ 10 mm (16.47%), and 1,136 cases with CPs diameter < 10 mm (83.53%). A total of 619 CPs patients with Hp infection had multiple polyps (n ≥ 2) (45.5%), and 741 cases had single polyp (n < 2) (54.5%). The Pearson χ^2^ test indicated that, compared with Hp-positive patients, Hp-negative patients had a lower probability of having multiple polyps (n ≥ 2) or larger polyps (diameter ≥ 10) (*P* < 0.05) (**Table 4**).

**Table 4:** The χ^2^ test for the effect of HP, and size, number of CPs.

Factor	HP (negative) n=3609	HP (positive) n=1360	χ^2^	*P* value
diameter≥10 mm	503	224	5.075	0.024**
diameter<10 mm	3106	1136		
number≥2	1480	619	8.221	
number <2	2129	741		0.004**

**P < 0.01.

**Figure 3:**
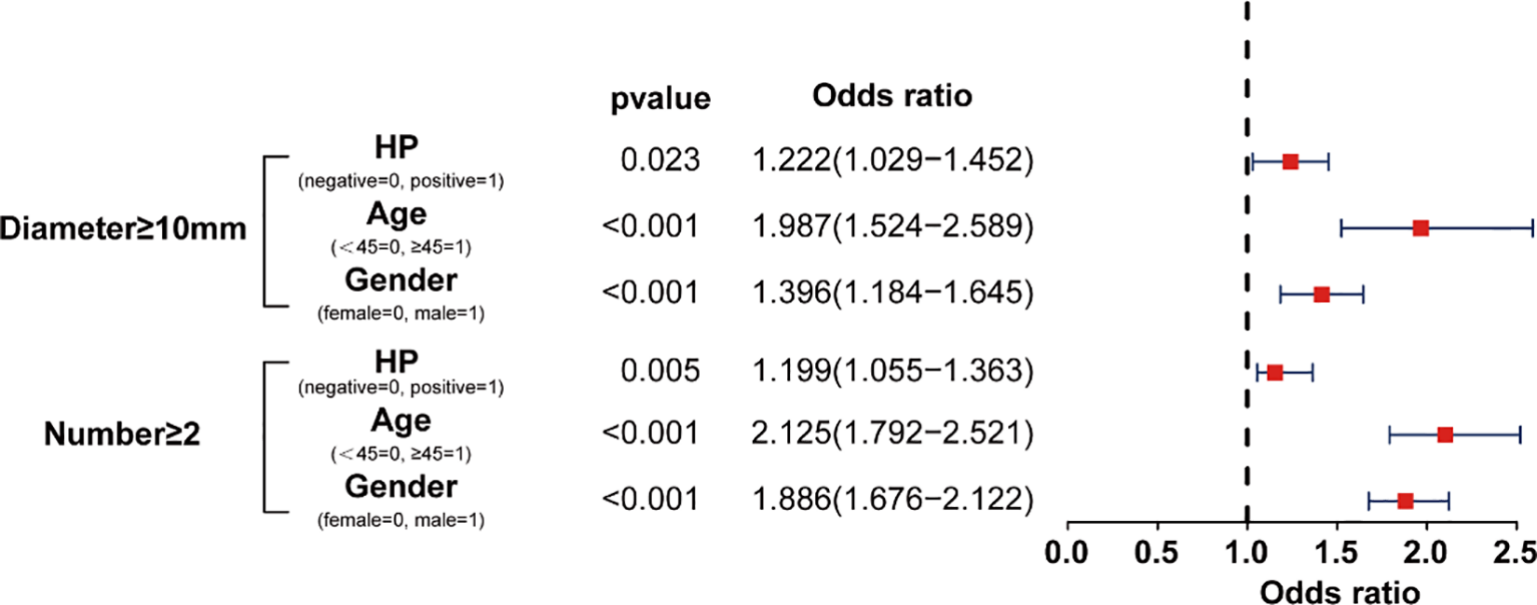
OR value of HP, gender, and age for the size and numbers of CPs.

Multivariate logistic regression analysis was used to determine whether Hp infection was related to the occurrence of polyp diameter ≥ 10 mm or multiple polyps (n ≥ 2). The results showed that the OR value was 1.222 (95%CI 1.029-1.452) for the incidence of CPs with a diameter ≥ 10 mm. The OR value is 1.199 (95%CI 1.09-1.40) for multiple CPs (n ≥ 2) (**Figure 3**). The OR values indicate that Hp is a risk factor for the incidence of multiple polyps and larger polyps.

### The Relationship Between Hp and Adenomas

Among the 4,969 cases in the CPs group, 4,435 cases were underwent biopsy. According to the pathological classification, there were 1,938 cases of hyperplastic polyps accounting for 43.7% and 2,497 cases of adenomas accounting for 56.3%. In the 3,211 Hp-negative patients, 1,396 cases were polyps accounting for 43.5%, and 1,815 cases were adenomas accounting for 56.5%. Among the 1,224 Hp-positive patients, 542 cases were hyperplastic polyps accounting for 44.3%, and 682 cases were adenomas accounting for 55.7%. The χ^2^ test showed no significant difference between Hp infection and hyperplastic polyps or adenomas (*P* > 0.05).

In addition, the 2,497 cases of adenomas were divided into four categories: tubular adenoma, serrated adenoma, villous adenoma, and mixed adenoma according to the pathological features. There were 1,811 Hp-negative cases, including 1,354 (74.8%) cases with tubular adenomas, 8 (0.4%) cases with serrated adenomas, 3 (0.2%) cases with villous adenomas, and 446 (24.6%) cases with mixed adenomas. A total of 686 Hp-positive patients included 505 (73.6%) cases with tubular adenomas, 5 (0.8%) cases with serrated adenomas, 3 (0.4%) cases with villous adenomas, and 173 (25.2%) cases with mixed adenomas. The results of χ^2^ test showed no statistically significant difference between Hp and the pathological type of adenoma (*P* > 0.05).

Furthermore, the 2,497 cases of adenomas were divided into two categories: serrated adenoma and nonserrated adenoma. There were 1,811 Hp-negative cases, including 4 (1.9%) serrated adenoma cases and 1,777 (98.1%) other adenoma cases. A total of 686 cases with Hp-positive included 19 (2.8%) cases with serrated adenoma and 667 (97.2%) cases with nonserrated adenoma. There was no statistically significant difference between Hp and serrated adenoma incidence (*P* > 0.05).

According to the pathological grade of neoplasia within adenoma, the adenomas are divided into high-grade internal neoplasia and low-grade internal neoplasia. A total of 1,811 Hp-negative cases included 156 (8.6%) high-grade internal neoplasia cases and 1655 (91.4%) low-grade internal neoplasia cases. A total of 686 Hp-positive cases were grouped into 66 (9.6%) cases with high-grade internal neoplasia and 620 (90.4%) cases with low-grade internal neoplasia. The χ^2^ test showed that there was no significant difference between Hp infection and neoplasia in adenoma (*P* > 0.05).

The above analysis indicated that Hp does not affect the incidence of adenoma or the pathological morphology or grade of adenoma.

### The Relationship Between Hp and the Results of Immunohistochemistry

Immunohistochemical examinations of P53, Ki-67, MLH1, PMS2, MSH2, and MSH6 were performed on 375 adenoma cases. According to the χ^2^ test, the positive rate of P53 nuclei in the immunohistochemistry of Hp-positive patients was significantly higher than that of Hp-negative patients (*P* < 0.05). There was no statistical significance between Hp and Ki-67 infection (*P* > 0.05) and the loss of mismatched repair proteins (*P* > 0.05) (**Table 5**). Studies have confirmed that P53 is a vital gene leading to CRCs susceptibility, and it may also be a crucial gene associated with CPs and early-onset of CRCs (15, 16). In addition, the higher the positive rate of P53 is, the higher the malignancy and recurrence rate of CPs are (17). The results of immunohistochemistry and χ^2^ **** analysis proved that Hp infection is related to the expression of P53, suggesting that Hp may affect the occurrence of CPs and CRCs by controlling the expression of P53.

**Table 5:** The effect of HP infection on the results of immunohistochemistry with χ^2^ test.

Immunohistochemistry		HP-negative (n=268)	HP-positive (n=107)	χ^2^	*P* value
P53	≤5%	154 (57.5%)	60 (56.1%)	11.048	0.026*
	6%~25%	69 (25.7%)	26 (24.3%)		
	26%~50%	30 (11.2%)	12 (11.2%)		
	51%~75%	4 (1.5%)	8 (7.5%)		
	<75%	11 (4.1%)	1 (0.9%)		
Ki-67	<30%	60 (22.4%)	27 (25.2%)	0.348	0.556
	≥30%	208 (77.6%)	80 (74.8%)		
misplaced ribonuclear proteins	normal	251 (93.6%)	97 (90.7%)	1.032	0.31
	missing	17 (6.4%)	10 (9.3%)		

*P < 0.05.

### The Relationship Between Hp Infection Levels and CPs, and CRC

According to the degree of Hp infection, 3,291 Hp positive cases were divided into Hp (+), Hp (++) and Hp (+++) groups. There were 2,469 Hp (+) cases, which included 1048 cases of CPs (42.44%); 445 Hp (++) cases, including 183 cases of CPs (41.12%); 377 Hp (+++) cases, including 167 CPs cases (44.30%). The results of χ2 test showed no significant difference between the degree of Hp infection and CPs (*P* > 0.05). In addition, among 2,469 Hp (+) cases, there were 27 CRC cases (10.94‰); 445 Hp (++) cases included 5 cases of CRC (11.23‰); and 377 Hp (+++) cases included 6 cases of CRC (15.91‰). The results of χ2 test showed no significant difference between the degree of HP infection and CRC (*P* > 0.05). The results show that the degree of Hp infection does not affect the incidence of CPs and CRC (**Table 6**).

**Table 6:** Analysis of Hp infectious levels.

Factor	NCPs n=1893	CPs n=1398	χ^2^	*P* value
HP(+)	1421	1048	0.846	0.0655
HP(++)	262	183		
HP(+++)	210	167		
**The χ^2^ test for the degree of Hp infection and CPs, CRC.**			
**Factor**	**NCRC n=3253**	**CRC n=38**	**χ^2^**	***P* value**
HP(+)	2442	27	0.715	0.6995
HP(++)	440	5		
HP(+++)	371	6		

### The Interaction of Hp, Age, and Gender for CPs

**Figure 4:**
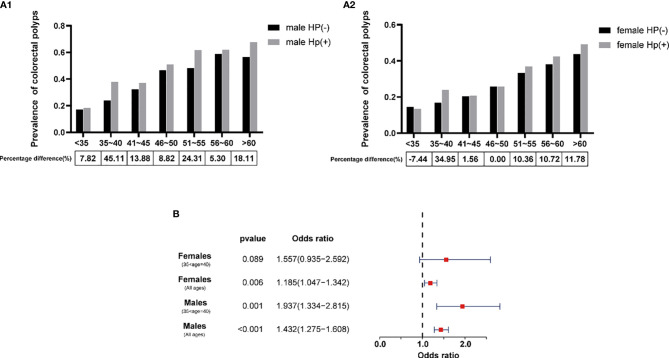
The influence of Hp on the prevalence of CPs in the Chinese of different ages. **(A1)** Relationship between the prevalence of CPs and Hp in males. **(A2)** Relationship between the prevalence of CPs and Hp in females. **(B)** OR value of Hp and different groups of age for CPs from logistic regression.

Age, gender, and Hp are recognized as risk factors for CPs. However, the impact of the combination of age, sex, and Hp infection on CPs is still unclear. To more accurately study the interaction of age, gender, and Hp infection on CPs, the cases aged 35 to 60 were specifically subdivided: < 35 years old, 36 to 40 years old, 41 to 45 years old, 46 to 50 years old, 51 to 55 years old, 56 to 60 years old, and > 60 years old. The secondary grouping was based on gender. We used multifactor variance analysis to discover the interaction of Hp infection and CPs in different ages and sexes. The percentage difference was calculated to reflect the impact of Hp infection on the prevalence of CPs (percentage difference = (Hp (+) - Hp (-))/((Hp (+) - Hp (-))/2) *100%). The results showed that, at the ages of 35-40, the prevalence of CPs in male Hp-positive patients was significantly higher than that in Hp-negative patients (45.11%) (**Figure 4A1**). The Hp-positive female patients aged 35-40 years had a significantly increased prevalence of CPs (increased by 34.95%) (**Figure 4A2**). The results suggest that we should further study the relationship between Hp and the prevalence of CPs in 35 to 40-years-old patients.

According to the results of logistic regression analysis, the OR value of Hp infection for CPs was 1.432 (95%CI 1.275-1.608) for all male cases. However, for males aged 35 to 40, the OR value of Hp suddenly increased to 1.937 (95%CI 1.334-2.815). The OR value of Hp for CPs was 1.185 (95%CI 1.047-1.342) for all female cases. For females aged 35 to 40, the OR value of Hp increased to 1.557 (95%CI 0.935-2.592), but the *P* value was more significant than 0.05 (**Figure 4B**). The results show that Hp is a high-risk factor for CPs in all age groups, regardless of sex. Among them, the OR value of Hp in Chinese patients aged 35-40 will increase significantly.

## Discussion

In China, CRC is the fifth most commonly diagnosed cancer and the fifth most common cause of cancer-inducing death (18). The incidence and mortality of CRCs in China are on the continuous rise, and the prevalence and mortality in urban areas are higher than those in rural areas (19). Most CRC comes from preexisting CPs (20), especially adenomas and serrated polyps (21). The concern for CPs cannot be ignored.

Screening for CPs and CRC-related risk factors is essential to prevent the prevalence of CPs and even CRCs. Since Hp has been identified as a significant cause of gastritis, gastric ulcer, and gastric cancer, it has received increasing scientific research attention (22). Scholars have gradually realized the relationship between Hp, CPs, and CRC (5–7). However, few large-sample clinical research of Chinese individuals directly clarifies it. Due to the persistently high Hp infection rate in China, it is imperative to confirm whether Hp is a risk factor for CPs and CRC as to the Chinese population and study the relationship between Hp, CPs, and CRC through analyzing Chinese patients’ gastrointestinal endoscopy data. A large volume of clinical cases and strong pertinence (for Chinese individuals only) are the unique features of the study.

In the 13,037 cases, the Hp infection rate was 25.2%, of which the female infection rate was 23.6%, and the male infection rate was 26.7%, indicating that the Hp infection rate in males may be slightly higher than that in females. This result is consistent with other’ research results (23), suggesting that men are more likely to be infected with Hp. In 9,746 Hp -negative cases, there were 3,609 cases with CPs (37.0%). In 3,291 Hp -positive cases, there were 1,360 cases with CPs (41.3%). Combined with the results of Pearson χ2 test, we found that Hp-positive patients were at higher risk of developing CPs. Multiple logistic regression analysis results showed that Hp is a risk factor for CPs and CRC. The OR values of Hp infection with CPs and CRC were 1.228 (95%CI 1.130-1.336) and 1.862 (95%CI 1.240-2.796), respectively. OR value greater than 1 means that Hp positive will increase the prevalence of CPs and CRC. Currently, some studies link Hp to CRC and prove that Hp is a high-risk factor for CRC (24), which is consistent with our results. However, few studies have linked Hp with CPs and the malignancy rate of CPs. The infection of CPs causes approximately 70% of CRC. CPs are vital indicators for early screening of CRC (25). We believe that discussing the relationship between Hp and CRC alone is somewhat inadequate. Hp -CPs-CRC should be analyzed systematically. Our results showed that Hp is a risk factor for both CPs and CRC, further supporting the importance of systematic analysis of the relationship between Hp-CPs-CRC. In addition, for Hp positive patients, the probability of multiple CPs (n ≥ 2) (OR: 1.199, 95% CI 1.09-1.40) and large-diameter polyps (diameter ≥ 10 mm) (OR: 1.222, 95% CI 1.029-1.452) was increased. Many studies have fully proven that multiple CPs and large-diameter CPs are more likely to transform into CRC than ordinary polyps (26). In the study, we found that Hp-positive patients with CPs had a higher P53 positive rate. The mutation and upregulated expression of P53 promote the rate of CPs malignancy by accelerating cell proliferation, and inhibiting cell apoptosis (27). The higher the expression of P53 is, the greater the recurrence and malignancy rate of CPs are in patients (28). Based on the above results, we thought that Hp positive might activate the expression of P53 and promote the incidence of CPs, especially multiple CPs and large-diameter CPs. The above CPs are likely to become CRC. This crucial correlation may also be a possible reason for Hp to be seen as having a high risk of CRC. Therefore, we have reasons to believe that Hp is a crucial risk factor for both CPs and CRC and suggest that Hp-positive patients should undergo colonoscopy in a timely manner.

However, in the study, we found that the degree of Hp infection did not affect the incidence of CPs and CRC. We believe that Hp might act as a ‘starting gun’ in the pathogenesis of CPs and CRC. Positive Hp rapidly activates the cyclooxygenase 2 (COX-2) and increases the expression of P53, thus increasing the incidence of CPs and CRC (29, 30). In this process, the presence/absence of Hp may play a more critical inducing role than the degree of Hp infection. The selected biopsy site under gastroscopy may also influence the above results. Antrum lesser curvature, upper body lesser curvature (UBLC) and upper body greater curvature (UBGC) are the main biopsy sites of the stomach. The study confirmed that the UBGC side is the most sensitive and specific biopsy site to detect Hp, where the Hp content may be higher (31). Meanwhile, the process of checking Hp under the gastroscope is to pick up a part of the gastric mucosa (biopsy site) for HE staining and observe whether Hp exists under the microscope. The degree of Hp infection was classified according to the number of Hp bacteria visible under the microscope. Although this detection method is the gold standard for clinical diagnosis of Hp, the reliability of the classification of Hp infection degree obtained by only detecting part of the gastric mucosa is not enough. The results obtained only represent the Hp infection at the biopsy site. Therefore, using the results of pathological detection of Hp to classify the degree of Hp infection is less reliable. In response to this shortcoming, we planned to use 13C-urea breath tests to detect the degree of Hp infection, quantify the degree of Hp infection, and determine the relationship between the degree of Hp infection and the prevalence of CPs and CRC.

Age and sex are currently recognized as high-risk pathogenic factors for CPs (32). To further research the interaction between age, gender, and Hp, we subdivided the cases into 7 age groups and conducted a multifactor analysis of variance for men and women separately. It was surprising to find that at the age of 35-40, whether males or females, Hp infection had a significant increase in the incidence of CPs. The OR value of Hp was 1.432 (95%CI 1.275-1.608) for all male cases and 1.937 (95%CI 1.334-2.815) for the males aged 35 to 40. The OR of Hp was 1.185 (95%CI 1.047-1.342) for all female cases. For females aged 35 to 40, the OR value of Hp increased to 1.557 (95%CI 0.935-2.592). Therefore, we proposed that compared to patients of other age groups, Chinese individuals aged 35-40 with Hp positivity should undergo colonoscopy regularly. It is vital to detect precancerous polyps through colonoscopy in time and remove them directly during the operation to reduce CRCs incidence (33).

## Conclusion

(1) Hp positivity will promote the incidence of CPs and CRC. However, the degree of Hp infection will not affect the incidence; (2) Hp-positive patients are prone to develop multiple polyps (n ≥ 2) and larger polyps (diameter > 10 mm); (3) Hp positivity has an impact on P53 expression in adenomas which may promote adenomas conversion to CRCs but has no effect on the formation and pathological morphology of adenomas; (4) The prevalence of CPs in Hp-positive Chinese patients aged 35-40 is significantly higher than that in other age groups, suggesting that Chinese individuals aged 35-40 with Hp positivity should undergo colonoscopy regularly.

## Data Availability Statement

The datasets presented in this article are not readily available because the datasets used and/or analyzed during the current study are available from the corresponding author on reasonable request. Requests to access the datasets should be directed to SX, xsmdoctor@163.com.

## Ethics Statement

The studies involving human participants were reviewed and approved by The Medical Ethics Committee of The First Affiliated Hospital of Zhejiang Chinese Medical University. Written informed consent for participation was not required for this study in accordance with the national legislation and the institutional requirements.

## Author Contributions

SX and SZ had full access to all the data in the study and took responsibility for the data integrity and data analysis accuracy. Concept and design: SX and SZ. Acquisition, analysis, or interpretation of data: CW, JY, SX, and SZ. Drafting of the manuscript: JY, SX, CW, and BH. Statistical analysis: CW and JY. All authors contributed to the article and approved the submitted version.

## Funding

This work was supported by Natural Science Foundation of Zhejiang Province (No.LY21H270009); Chinese Medicine Science and Technology Plan of Zhejiang Province (Grant number: 2020ZB065); The Medicine and Health Science and Technology Plan Projects in Zhejiang province (Grant number: 2019RC057); Key research projects of traditional Chinese medicine in Zhejiang Province (No.2020ZZ007); Natural Science Foundation of China (No.815737603).

## Conflict of Interest

The authors declare that the research was conducted in the absence of any commercial or financial relationships that could be construed as a potential conflict of interest.

## Publisher’s Note

All claims expressed in this article are solely those of the authors and do not necessarily represent those of their affiliated organizations, or those of the publisher, the editors and the reviewers. Any product that may be evaluated in this article, or claim that may be made by its manufacturer, is not guaranteed or endorsed by the publisher.

## Abbreviations

HP, Helicobacter pylori; COX-2, Cyclooxygenase 2; CPs, Colorectal polyps; CRC, Colorectal cancer; NCPs, non Colorectal polyps; OR, Odds ratio; CI, Confidence Interval; UBLC, Upper body lesser curvature; UBGC, Upper body greater curvature.
